# Development of a frailty index from the Dutch public health monitor 2016 and investigation of its psychometric properties: a cross-sectional study

**DOI:** 10.1186/s13690-023-01093-4

**Published:** 2023-04-28

**Authors:** Nanda Kleinenberg-Talsma, Fons van der Lucht, Harriët Jager-Wittenaar, Wim Krijnen, Evelyn Finnema

**Affiliations:** 1 Research Group Healthy Ageing, Allied Health Care and Nursing, Hanze University of Applied Sciences, Groningen, The Netherlands; 2 Department of Science in Healthy Ageing and Healthcare (SHARE), University Medical Center Groningen, University of Groningen, Groningen, The Netherlands; 3 FAITH research, Groningen/Leeuwarden, The Netherlands; 4 Centre for Health and Society, National Institute of Public Health and the Environment, Bilthoven, The Netherlands; 5 Aletta Jacobs School of Public Health, FAITH research, Groningen, The Netherlands; 6 Department of Oral and Maxillofacial Surgery, University Medical Center Groningen, University of Groningen, Groningen, The Netherlands; 7 Faculty of Physical Education and Physiotherapy, Department Physiotherapy and Human Anatomy, Research Unit Experimental Anatomy, Vrije Universiteit Brussel, Brussels, Belgium; 8 Faculty of Science and Engineering, University of Groningen, Groningen, The Netherlands; 9 Department of Health Science, Section of Nursing Research, University Medical Center Groningen, University of Groningen, Groningen, The Netherlands; 10 Research Group Nursing Diagnostics, Hanze University of Applied Sciences, Groningen, The Netherlands; 11 Research Group Living, Wellbeing and Care for Older People, NHL Stenden University of Applied Sciences, Leeuwarden, The Netherlands

**Keywords:** Frailty, Health monitors, Psychometric analysis, Older adults

## Abstract

**Background:**

Frailty in older adults is an increasing challenge for individuals, health care organizations and public health, both globally and in The Netherlands. To focus on frailty prevention from a public health perspective, understanding of frailty status is needed. To enable measurement of frailty within a health survey that currently does not contain an established frailty instrument, we aimed to construct a frailty index (FI) and investigate its psychometric properties.

**Methods:**

We conducted a cross-sectional study using data from the Dutch Public Health Monitor (DPHM), including respondents aged ≥ 65 years (n = 233,498). Forty-two health deficits were selected based on literature, previously constructed FIs, face validity and standard criteria for FI construction. Deficits were first explored by calculating Cronbach’s alpha, point-polyserial correlations, and factor loadings. Thereafter, we used the Graded Response Model (GRM) to assess item difficulty, item discrimination, and category thresholds.

**Results:**

Cronbach’s alpha for the 42 items was 0.91. Thirty-seven deficits showed strong psychometric properties: they scored above the cutoff values for point-polyserial correlations (0.3) or factor loadings (0.4) and had moderate to very high discrimination parameters (≥ 0.65). These deficits were retained in the scale. Retaining the deficits with favorable measurement properties and removing the remaining deficits resulted in the FI-HM37.

**Conclusion:**

The FI-HM37 was developed, an FI with 37 deficits indicative of frailty, both statistically and conceptually. Our results indicate that health monitors can be used to measure frailty, even though they were not directly designed to do so. The GRM is a suitable approach for deficit selection, resulting in a psychometrically strong scale, that facilitates assessment of frailty levels using the DPHM.

## Introduction

The proportion of older adults in the population is increasing globally, and will continue to grow in the coming years [1]. In for instance the Netherlands, expectations are similar: by 2040, 30% of the Dutch population, i.e., 4.7 million people, is expected to be 65 years of age or over [2]. A higher age indicates a higher risk of frailty, and consequently, the prevalence of frail older adults in the population is also increasing [3].

Frailty can be considered as a dynamic process in which one experiences a decline in a single or in several health domains (i.e., physical, psychological or social), which in turn increases the risk of adverse health outcomes [4]. In other words, frailty can be considered as increasing numbers of deficits in health, impacting on other negative health outcomes. This poses a great challenge to the wellbeing and quality of life of individuals, as well as to public health [3, 5]. However, as indicated in Gobbens’ definition, frailty is seen a dynamic process, and someones frailty level can be placed on a continuum ranging from not frail to very frail [4]. Hence, up to a certain point, frailty in older adults can be prevented, and in early stages even reversed [3]. Therefore, early detection and prevention of frailty in older adults is of great importance.

To focus on frailty prevention from a public health perspective, first it is necessary to have a thorough understanding of the frailty status in older adults. Information about frailty levels, knowing which groups in the population are frail or face the risk of becoming frail, can inform preventive policy and action [6, 7]. National health surveys collect a variety of health-related information for purposes such as monitoring trends in population health, assessing the prevalence of disease or health care use [8, 9]. As in many countries, in the Netherlands a national health survey is held every four year since 2012. More specifically, the Dutch Public Health Monitor (DPHM)[10] collects a wide range of topics related to self-reported health, making it a rich source of information. However, existing frailty instruments are included neither in the DPHM, nor in other European health monitors.

Despite the absence of an established frailty instrument in existing health surveys, these surveys do include many symptoms and topics that in fact underlie the concept of frailty. Since many of these symptoms and topics represent deficits in different health domains, this offers the opportunity to explore the possibility of frailty measurement based on the accumulation of health deficits, by means of a frailty index (FI). An FI is a way to operationalize frailty in accordance with the frailty concept as described by Gobbens [4], by encompassing a range of health deficits in multiple domains (e.g., physical, social, psychological). Besides, it allows for the assessment of overall frailty including several frailty domains, and for comparisons across populations and environments [11, 12]. Health deficits, which may include signs, symptoms, disabilities or diseases [13], are selected and their presence is counted. The FI is intended to be used as a continuous score [14], with the more deficits present, the higher the level of frailty. The procedure for creating an FI was first developed by Kenneth Rockwood and Arnold Mitnitski in 2001, and has been described thoroughly [13, 15]. This led to a multitude of studies in which FIs have been constructed and validated in different data sets, both in the Netherlands as well as in other European countries [11, 16–19]. FIs were often developed in existing datasets, such as the Swiss RAI-HC MDS [17] or the Longitudinal Aging Study Amsterdam (LASA) [16]. They are composed of different numbers of health deficits, ranging from 32 to 52, and include multiple health domains, e.g., physical activity, (self-rated) health, cognition, emotion/mood and nutrition [11, 16–19].

However, to the best of our knowledge, in Europe, no attempts have been made to operationalize frailty in older adults with the data of the national health monitors, although it provides a good opportunity for population measurement. Constructing a frailty index from a national health monitor could serve as a basis for epidemiologists, policymakers and other public health workers, e.g. to compare the degree of frailty of different groups in the population or in specific regions or neighbourhoods. Furthermore, few studies have meticulously investigated the psychometric properties of the separate health deficits to be included in an FI [20]. Even though FIs can be constructed from different deficits and different numbers of deficits [15], methods from Item Response Theory (IRT) provide detailed information about the separate health deficits in a scale, making it possible to assess each deficits’ contribution [21, 22]. In this way, psychometric analysis can contribute to understanding to what extent health deficits are indicative of frailty.

To bridge this gap in current frailty research, we intended to develop an FI from an existing health monitor and to use detailed psychometric methods to thoroughly investigate the scale and its separate health deficits.

## Materials and methods

### Study population

A cross-sectional study was conducted using data from the Dutch Public Health Monitor (DPHM) in the Netherlands. The DPHM consists of a survey which is administered every four years by the Community Health Services, in collaboration with Statistics Netherlands and the National Institute for Public Health and the Environment. The purpose of the survey is to collect health-related information and determinants of health, as well as the social situation and lifestyle of Dutch citizens aged ≥ 19 years [10, 23]. The DPHM is comprised of the Health Monitor for adults (≥ 19–64 years of age) and for older adults (≥ 65 years of age) (both conducted by the Community Health Services). Respondents were approached by means of an invitation letter and asked to fill out the survey online. In some regions of the Community Health Services, a paper version of the survey was enclosed in the invitation letter, while in others, the paper survey was only sent in case of non-response to the request for online participation. Finally, 0.1% of the surveys was collected by face to face interviews or via telephone. For the DPHM of 2016, information of 457,153 Dutch participants from private households was collected in all 25 regions of the Community Health Services. The response rate was around 40%. For the current study, the respondents aged ≥ 65 years were selected (n = 233,498). More information about procedures and construction of the survey has been described elsewhere [10, 23].

### Patient and public involvement

The current study is a cross-sectional study analyzing secondary data. Patients or public were not directly involved in the design of the study, in data analysis and reporting, nor will there be in dissemination plans for the research.

### Selection of health deficits

To construct an FI, the procedures as described previously were followed [13, 15]. An FI can be represented as a proportion, in which the number of deficits present in a person is divided by the total number of selected health deficits. The more deficits present, the more likely one is to be frail [15]. For a stable and an accurate index, it is necessary to include approximately 30 deficits from various health domains, following several specified criteria [15, 18].

**Fig. 1:**
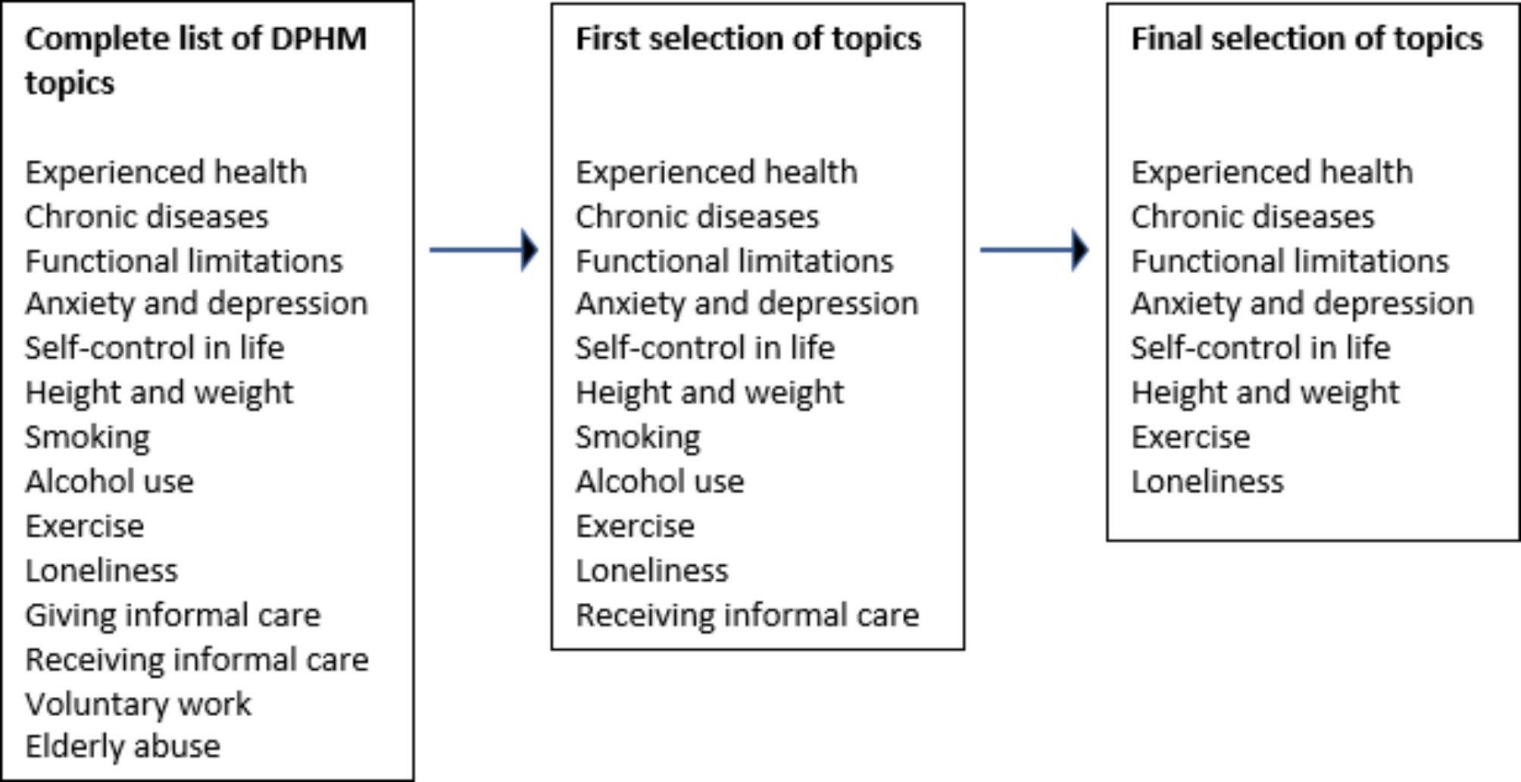
The selection process of health deficits from the Dutch Public Health Monitor

The DPHM consists of questionnaire items related to socio-demographic information (e.g., age, education, financial situation), health and health experiences (e.g., chronic conditions, disability, anxiety and depression), lifestyle factors (e.g., smoking, exercise) and social situation (e.g., loneliness). For the selection process, the DPHM topics were discussed by the main researchers (NK and FvdL). Most topics consisted of several items, e.g. the topic ‘chronic conditions’ consists of two separate items about the presence and the impact of chronic conditions, and the topic ‘alcohol use’ consists of seven items about the type and amount of alcohol used. Some of the topics constisted of existing scales, e.g. the topic ‘loneliness’ consisted of the 11 items of the De Jong Gierveld scale for emotional and social loneliness [24]. Other topics, however, such as ‘experienced health’ or ‘voluntary work’ consisted of one item. After discussing the complete list of DPHM topics, a first selection of items was made by the main researcher (NK). A list of 46 items was then discussed with the full research team, leading to the rejection of several items. Items were selected based on literature [13], items from previous FI construction [11, 16, 18, 19, 25], face validity, and the criteria advised by Searle et al., (2008) such as being related to health status or generally increasing with age [15]. Specifically, rejected items were, for example, items that were not directly relating to health status or age (e.g. elderly abuse or doing voluntary work) or items that represented lifestyle factors but not health outcomes (e.g., smoking or alcohol use). Included items were related to health status, were commonly used in other FIs [11, 16, 18, 19, 25], and were from different health domains: the physical, psychological and social domain, in line with Gobbens’ definition of frailty [4]. The remaining 42 items were investigated further. In Fig. 1, the process of deficit selection is presented.

Item response categories were coded into increasing numbers between zero and one. That is, binary items received values 0 or 1 (e.g. Do you have one or more chronic conditions – yes; no); variables with three categories received values 0, 0.5 or 1 (e.g. There are many people I can trust – yes; more or less; no); variables with four categories received values 0, 0.33, 0.66, or 1 (e.g. Are you able to bend and lift – yes, without difficulty; yes, with some difficulty; yes, with much difficulty; no); and variables with five categories received values 0, 0.25, 0.5, 0.75, or 1 (e.g. How often in the past 4 weeks did you feel everything is an effort – never; rarely; sometimes; often; always). Some deficits are formulated in an opposite direction. These were reversely coded to ensure a positive association with the frailty concept. Thus hypothetically, the higher the score, the more the deficit is present.

The recoding of BMI was somewhat more complex due to differences in optimal BMI values for adults aged ≥ 70 years [26]. BMI was calculated as weight x weight / height, resulting in kg/m^2^. For adults from 65 to 69 years, BMI < 20 kg/m^2^ is considered underweight, BMI 20 to < 28 kg/m^2^ as normal weight, BMI 28 to < 30 kg/m^2^ as overweight, and BMI > 30 kg/m^2^ as obese. For adults of ≥ 70 years, optimal BMI values are slightly different, therefore, BMI < 22 kg/m^2^ is considered underweight, BMI 22 to < 28 kg/m^2^ as normal weight, BMI 28 to < 30 kg/m^2^ as overweight, and BMI > 30 kg/m^2^ as obese [26]. Normal weight was recoded into 0, overweight into 0.5, and obese or underweight was recoded into 1.

Physical activity was included in a way that specifically aimed at the older population. In the DPHM, physical activity was assessed by the Short Questionnaire to Assess Health Enhancing Physical Activity (SQUASH) from which we selected three items about adherence to the Dutch Guideline for physical activity (PA), i.e., time spent on physical activity (weekly); frequency of bone and muscle strengthening activities; and frequency of balance strengthening exercises. The latter is included in the Dutch PA guideline specifically for elderly people [27]. These items were recoded into 0 and 1, indicating adherence (0) or no adherence (1) to the Dutch Guidelines.

### Data analysis

After FI construction, frailty scores were calculated by dividing the present deficits by the total number of deficits for each of the participants, indicating the proportion of total deficits. Frailty scores were also calculated per domain. For this purpose, the proportion of deficits represents the FI of a specific domain. For each respondent, FI scores were only calculated when all items were completed [28].

We conducted psychometric analysis using a number of suitable packages in R [29]. Several measures were calculated to investigate the quality of the health deficits and the frailty scale: Cronbach’s alpha for internal consistency, and point-polyserial correlations and factor loadings to indicate the correlations between the deficits and frailty. Deficits with both a point-polyserial correlation below 0.3 and a factor loading below 0.40 were considered as critical [30, 31]. Cronbach’s alpha for the scale needs to be above 0.70 [32]. In addition, analysis by the Graded Response Model (GRM) was used [21, 33, 34], to provide detailed information about the deficits and their categories in the frailty scale under construction, e.g., information about item difficulty, item discrimination and category thresholds for each item [21, 22]. Item difficulty measures the proportion of respondents reporting a health deficit: some deficits and categories represent more severe health problems that will be reported by a smaller proportion of respondents, while other deficits represent less severe health problems that will be reported by a larger proportion of respondents [22]. Item discrimination concerns the slope or the steepness of the item. The GRM is able to handle polytomous data with different numbers of response categories [33]. For evaluation purposes the following cut-off scores were used for the discrimination parameters: <0.35 (very low), 0.35–0.64 (low), 0.65–1.34 (moderate), 1.35–1.69 (high), and > 1.70 (very high) [35].

An important requirement is that category thresholds for each health deficit should be increasing monotonically, i.e., the higher one scores, the more likely one is to be frail. The GRM provides information about the category thresholds, which cannot be identified using Cronbach’s alpha. Monotonically increasing deficits also provide an indication that categories are well understood and used by the respondents as intended in the survey. For weak items, the increase in thresholds is expected not to be monotonically.

Health deficits scoring below the cut-off values for all three criteria, i.e., point-polyserial correlations, factor loadings, and having low or very low discrimination parameters were deselected in order to construct a suitable FI scale. Data summaries are presented as mean ± SD in the case of the normal distribution and as median and IQR in case of non-normality.

## Results

Demographics of the sample of participants are shown in Table 1. The total sample consists of 233.498 respondents, with slightly more women (52%) than men (48%). The mean age was 73.7 years. The older the age groups, the smaller are the group sizes: 65–69 years of age is the largest group (33.8%), followed by 70–74 (26.3%), 75–79 (19.8%), 80–84 (12.3%) and finally the group of respondents aged ≥ 85 (7.9%).

**Table 1:** Basic demographics of the study sample taken from the Dutch Public Health Monitor 2016

Population group		n (%)
**All**		233,498
**Gender**	**Female**	121,472 (52)
	**Male**	112,026 (48)
**Age category, years**	**65–69**	78,899 (33.8)
	**70–74**	61,345 (26.3)
	**75–79**	46,123 (19.8)
	**80–84**	28,650 (12.3)
	**≥ 85**	18,481 (7.9)

This selection process for FI construction yielded 42 health deficits, encompassing the physical, psychological and social domains. The physical domain consists of 14 deficits in total. Here, we included seven deficits related to functioning from the OECD Long Term Disability Questionnaire [36], as well as seven deficits about self-rated health, physical condition, and physical activity. For the psychological domain, 17 health deficits were selected, consisting of the K10 scale for anxiety and depression, and the Pearlin Mastery scale, both being validated scales [37, 38]. Finally, the social domain consists of a total of 11 deficits, including the De Jong Gierveld Scale, which is a validated scale for social and emotional loneliness [24]. The proportion of answers of the respondents per deficit category, Cronbach’s alpha, point polyserial correlations and factor loadings are presented in Table 2. Thirty-seven health deficits passed all of the above criteria of point polyserial correlations and factor loadings and five deficits were critical. In the physical domain, these were BMI and three deficits regarding adherence to physical activity guidelines: “minutes per week spent on moderate physical activity” (i.e., activities with moderate intensity, such as walking or cycling), “bone and muscle strengthening activities”, and “balance exercises”. In the psychological domain, the critical deficit was “a sense of control over one’s own future”. The social domain had two deficits with somewhat lower loadings: “being able to talk about daily problems” (0.40) and “having many people to trust” (0.37). However, these items had point-polyserial correlations larger than 0.3. Cronbach’s alpha for the 42 deficits was 0.91 and well beyond the threshold of 0.70.

**Table 2:** Psychometric properties of the 42-item Frailty Index, including item description, proportions for response categories, α if item deleted, point-polyserial correlations and factor loadings

Deficits and Subdomains	Proportions per response category*					α if item deleted	Point polyserial correlation	Factor loadings
**Physical domain**	**1**	**2**	**3**	**4**	**5**			
In general, how would you rate your own health?	0.10	0.55	0.30	0.05	0.01	0.92	0.64	0.69
Do you have one or more chronic conditions?	0.52	0.48				0.93	0.38	0.47
Are you limited in activities because of your health?	0.50	0.43	0.07			0.92	0.60	0.66
Are you able to follow conversation with three or more?	0.67	0.25	0.05	0.02		0.93	0.39	0.44
Are you able to have conversation with one person?	0.92	0.07	0.01	0.00		0.93	0.36	0.55
Are you able to read small letters?	0.78	0.16	0.03	0.03		0.93	0.39	0.49
Are you able to recognize face?	0.89	0.08	0.02	0.01		0.93	0.36	0.52
Are you able to carry 5 kg?	0.71	0.16	0.05	0.08		0.92	0.60	0.68
Are you able to bend and lift?	0.72	0.19	0.06	0.03		0.92	0.59	0.66
Are you able to walk for 400 m?	0.75	0.12	0.04	0.08		0.92	0.59	0.68
Body Mass Index (BMI)	0.59	0.17	0.24			0.93	0.20	0.19
Adherence to guideline: minutes per week spent on moderate physical activity	0.44	0.56				0.93	0.29	0.35
Adherence to guideline: bone and muscle strengthening activities	0.73	0.27				0.93	0.25	0.30
Adherence to guideline: balance exercises	0.07	0.93				0.93	0.06	0.08
**Psychological domain**	**1**	**2**	**3**	**4**	**5**	**α if item deleted**	**Point polyserial correlation**	**Factor loadings**
How often in the past 4 weeks did you feel very tired without clear cause?	0.35	0.32	0.22	0.08	0.03	0.92	0.69	0.72
How often in the past 4 weeks did you feel nervous?	0.41	0.37	0.18	0.03	0.01	0.92	0.60	0.65
How often in the past 4 weeks did you feel so nervous that you could not calm down?	0.69	0.18	0.10	0.02	0.00	0.92	0.61	0.71
How often in the past 4 weeks did you feel hopeless?	0.74	0.15	0.08	0.02	0.01	0.92	0.70	0.83
How often in the past 4 weeks did you feel restless?	0.47	0.37	0.13	0.02	0.01	0.92	0.64	0.70
How often in the past 4 weeks did you feel so restless that you could not sit still?	0.74	0.17	0.07	0.01	0.00	0.92	0.56	0.67
How often in the past 4 weeks did you feel sad or depressed?	0.60	0.27	0.11	0.02	0.00	0.92	0.67	0.76
How often in the past 4 weeks did you feel everything is an effort?	0.48	0.32	0.13	0.05	0.02	0.92	0.76	0.81
How often in the past 4 weeks did you feel so sad that nothing helped?	0.76	0.15	0.07	0.01	0.00	0.92	0.69	0.83
How often in the past 4 weeks did you feel useless?	0.78	0.13	0.07	0.01	0.01	0.92	0.61	0.74
I feel I have little control over things that happen to me	0.29	0.37	0.16	0.13	0.05	0.92	0.63	0.63
I feel there is no way I can solve some of my problems	0.31	0.36	0.13	0.14	0.05	0.92	0.68	0.68
There is little I can do to change important things in my life	0.28	0.37	0.15	0.16	0.05	0.92	0.68	0.69
I often feel helpless in dealing with problems of life	0.36	0.39	0.13	0.09	0.02	0.92	0.73	0.75
I sometimes feel I am being pushed around in life	0.43	0.37	0.11	0.07	0.02	0.92	0.64	0.67
What happens to me in the future mostly depends on me	0.14	0.31	0.23	0.19	0.12	0.93	0.18	0.10
I can do about anything if I set my mind to it	0.14	0.37	0.24	0.17	0.08	0.93	0.41	0.36
**Social domain**	**1**	**2**	**3**			**α if item deleted**	**Point polyserial correlation**	**Factor loadings**
Can you talk about daily problems	0.70	0.23	0.07			0.93	0.34	0.40
Do you have people to lean on when having problems	0.69	0.23	0.08			0.93	0.37	0.43
There are many people I can trust	0.51	0.36	0.13			0.93	0.34	0.37
I have enough people I feel close to	0.67	0.24	0.09			0.93	0.36	0.41
I can rely on friends when I need them	0.65	0.26	0.09			0.93	0.42	0.47
I miss a really close friend	0.78	0.14	0.08			0.93	0.44	0.55
I experience emptiness	0.80	0.14	0.06			0.93	0.53	0.68
I miss the pleasure of company	0.77	0.16	0.06			0.93	0.53	0.66
My circle of friends is too limited	0.71	0.19	0.09			0.93	0.46	0.56
I miss having people around	0.78	0.15	0.06			0.93	0.50	0.64
I often feel rejected	0.87	0.09	0.04			0.93	0.49	0.70

* Item Response categories differed between two to five, where the lowest category [1] indicates absence of the health deficit, and the highest category indicates presence of the deficit

The results from the more detailed GRM analysis of the responses of the participants on the health deficits are shown in Table 3. All thresholds for the 42 deficits show a monotonical increase as shown by the increasing betas, confirming the intended ordering in the deficit categories.

**Table 3:** Graded Response Model results of the 42-item Frailty Index, including thresholds (difficulty) and slopes (discrimination parameter)

Physical domain	Thresholds (Difficulty)				Slope (Discrimination parameter)
	**beta.1**	**beta.2**	**beta.3**	**beta.4**	**beta**
In general, how would you rate your own health?	-3.190	0.856	3.882	6.487	1.571
Do you have one or more chronic conditions?	0.091				0.805
Are you limited in activities because of your health?	-0.046	3.382			1.412
Are you able to follow conversation with three or more?	0.777	2.705	3.965		0.735
Are you able to have conversation with one person?	2.887	5.008	6.153		1.116
Are you able to read small letters?	1.443	3.012	3.789		0.872
Are you able to recognize face?	2.517	3.965	4.769		1.026
Are you able to carry 5 kg?	1.251	2.597	3.259		1.504
Are you able to bend and lift?	1.274	3.029	4.435		1.404
Are you able to walk for 400 m?	1.549	2.671	3.282		1.476
Body Mass Index (BMI)	0.384	1.177			0.283
Adherence to guideline: minutes per week spent on moderate physical activity	-0.281				0.530
Adherence to guideline: bone and muscle strengthening activities	1.028				0.456
Adherence to guideline: balance exercises	-2.523				0.089
**Psychological Domain**	**Thresholds**				**Slope**
	**beta.1**	**beta.2**	**beta.3**	**beta.4**	**beta**
How often in the past 4 weeks did you feel very tired without clear cause?	-0.982	1.061	3.028	4.911	1.734
How often in the past 4 weeks did you feel nervous?	0.570	1.713	4.167	6.185	1.488
How often in the past 4 weeks did you feel so nervous that you could not calm down?	1.232	2.911	5.257	7.298	1.766
How often in the past 4 weeks did you feel hopeless?	2.046	4.006	6.678	8.546	2.583
How often in the past 4 weeks did you feel restless?	-0.220	2.407	4.835	6.860	1.714
How often in the past 4 weeks did you feel so restless that you could not sit still?	1.506	3.161	5.270	7.168	1.578
How often in the past 4 weeks did you feel sad or depressed?	0.623	2.986	5.569	7.556	2.041
How often in the past 4 weeks did you feel everything is an effort?	-0.158	2.514	4.502	6.549	2.355
How often in the past 4 weeks did you feel so sad that nothing helped?	2.238	4.283	6.986	9.116	2.626
How often in the past 4 weeks did you feel useless?	2.022	3.628	5.631	7.225	1.952
I feel I have little control over things that happen to me	-1.297	1.046	2.282	4.093	1.610
I feel there is no way I can solve some of my problems	-1.255	1.193	2.225	4.240	1.831
There is little I can do to change important things in my life	-1.515	1.006	2.176	4.429	1.838
I often feel helpless in dealing with problems of life	-1.036	1.990	3.564	5.862	2.220
I sometimes feel I am being pushed around in life	-0.483	2.083	3.426	5.366	1.734
What happens to me in the future mostly depends on me	-1.824	-0.174	0.822	2.037	0.241
I can do about anything if I set my mind to it	-2.046	0.087	1.273	2.642	0.736
**Social Domain**	**Thresholds**				**Slope**
	**beta.1**	**beta.2**			**beta**
Can you talk about daily problems	0.915	2.722			0.620
Do you have people to lean on when having problems	0.886	2.671			0.693
There are many people I can trust	0.020	1.992			0.552
I have enough people I feel close to	0.758	2.475			0.653
I can rely on friends when I need them	0.683	2.551			0.747
I miss a really close friend	1.485	2.777			0.992
I experience emptiness	1.923	3.599			1.444
I miss the pleasure of company	1.633	3.426			1.340
My circle of friends is too limited	1.075	2.618			0.963
I miss having people around	1.660	3.322			1.246
I often feel rejected	2.720	4.450			1.568

Thirty-five deficits showed moderate to very high discrimination parameters. In the physical domain, discrimination parameters for the following deficits were low to very low: BMI (0.283), three deficits regarding adherence to physical activity guidelines: “minutes per week spent on moderate physical activity” (0.530), “bone and muscle strengthening activities” (0.456), and “balance exercises” (0.089). In the psychological domain, the deficit “a sense of control over one’s own future” showed very low discrimination (0.241). Two deficits of the social domain showed low discrimination parameters: “being able to talk about daily problems” (0.620) and “having many people to trust” (0.552).

Based on these analyses, the quality of the health deficits was assessed. We removed the five deficits that scored below the cut-off values for both point-polyserial correlations and factor loadings and had low or very low discrimination parameters. Scale characteristics of the 37-item FI were slightly better than those of the 42-item FI. Cronbach’s alpha was 0.912 for the FI including 42 deficits, and 0.927 for the FI including 37 deficits. Likewise, for the Graded Response Model, log-likelihood of the 42-item scale was − 7,406,515, and − 6,534,305 for the 37-item scale.

Based on item scores between zero and one, the FI constructed from the Health Monitor was computed using the remaining 37 deficits, i.e., the FI-HM37. As presented in Table 4, the FI-HM37 results in an overall mean frailty score of 0.19 ± 0.14.

**Table 4:** Mean, median, and Inter quartile range of the overall 37-item Frailty Index and for the included domains

	N*	Mean ± SD	Median	IQR
**FI Total – 37 deficits**	181,350	0.19 ± 0.14	0.15	0.07
**FI Physical domain – 10 deficits**	215,063	0.19 ± 0.17	0.15	0.10
**FI Psychological domain – 16 deficits**	204,594	0.20 ± 0.16	0.17	0.08
**FI Social domain – 11 deficits**	206,196	0.18 ± 0.20	0.09	0.09

* Number of respondents differ due to the fact that FI was calculated for respondents without missing deficits. The complete sample consists of 233,498 respondents

In Table 5, more specific results from the FI-HM37 are presented. Frailty scores are higher in women then in men for overall frailty, as well as for the separate domains. Notably, the overall frailty scores as well as the domain scores systematically increase with increasing age. Tests for differences in mean scores showed that for gender and for all age groups, mean FI scores and mean domain scores statistically significantly differed from each other (*p* ≤ 0.01).

**Table 5:** Mean frailty scores based on the 37-item Frailty Index, by gender and age groups

Population group		FI overall	Physical domain	Psychological domain	Social domain
**Gender**	**Male**	0.17	0.17	0.18	0.17
	**Female**	0.20	0.20	0.23	0.19
**Age category, years**	**65–69**	0.16	0.14	0.18	0.16
	**70–74**	0.17	0.16	0.19	0.17
	**75–79**	0.20	0.19	0.22	0.19
	**80–84**	0.24	0.26	0.25	0.21
	**≥ 85**	0.30	0.35	0.29	0.26

## Discussion

In this study, we developed the FI-HM37 based upon psychometrically strong health deficits, with the data of a large national health survey in the general Dutch population, which did not yet contain an established frailty instrument. We showed that it is possible to use a national health survey to measure frailty levels in the Dutch older population, based on a deficit accumulation approach. Out of 42 preselected deficits, 37 contributed sufficiently to measuring the concept of frailty. Exposing five deficits with weak psychometric properties strongly suggests the importance of deficit selection during FI construction in order to understand to what extent health deficits are indicative of frailty.

The current study using the Dutch Public Health Monitor facilitates measurement of frailty in the older home-dwelling population in The Netherlands. Since the DPHM is an existing survey of which the standard items are used to measure frailty, there is no additional burden for respondents. The FI-HM37 is not developed to be employed as a separate instrument, but provides an additional application of the DPHM, increasing its usability. Previously, items included in regional subdivisions of the DPHM have been used to derive frailty indices regionally [25, 39]. These initiatives yielded estimations of frailty for two of the 25 regions, based on different frailty instruments. However, frailty measurement on the basis of the nationwide included items in the DPHM had not been conducted before. In doing so, this study adds to the need of gaining insight in the frailty concept on population level.

The mean FI score of 0.19 observed in the current study is somewhat higher than the findings from two non-European studies in which an FI was developed from national health surveys: a recent study in Chile, where a mean FI score of 0.15 was found [28] and a Brazilian study in which a mean FI score of 0.13 was found [40]. Differences are possibly due to different ages of the population: our sample consisted of adults aged ≥ 65, while the Chilean and Brazilian samples consisted of adults aged ≥ 40 and ≥ 60, respectively [28, 40].

Similar to previously developed FIs [15, 16], scores on the FI-HM37 increase with age and mean scores are higher in women than in men, providing a first indication for construct validity.

Furthermore, the study psychometrically assessed the health deficits used in the FI-HM37 by means of the GRM. Although the procedures for FI development allow flexibility in deficit choice and number [15], using detailed psychometric methods ratifies the selection process of health deficits, by including only those deficits that contribute most to the measurement of the concept. Among the methods for item response assessment, Widagdo et al. were the first to use Rasch analysis for dichotomous items to assess the construct validity of an FI [20]. The GRM used in the current study is a generalization of the Rasch model, suitable for items with mixed numbers of ordered categories, providing detailed information about item properties and thereby making selection of health deficits better feasible. More specifically, the item thresholds from GRM revealed the order in the item categories, indicating that categories were well understood by the respondents as intended during survey construction. Furthermore, item thresholds provided information about the position of each item category on the latent frailty trait. The variation in positions showed that the continuum of frailty is being covered with both easy to endorse as well as more difficult to endorse deficits [35] which reflect different severities within deficits.

Several strengths of this study deserve mentioning. First, the DPHM was used, an existing health-related survey, that has not yet been used for determining frailty levels in older adults on national level. As mentioned earlier, the existing collection of DPHM deficits can be used as a basis for measuring frailty [13]. By using the survey for this purpose, additional means of application on national level were established for the DPHM. Second, the dataset is derived from a very extensively weighed sample. All Dutch municipalities were included, making the selected sample of participants highly representative for the population [23]. Third, applying the GRM to the development of an FI is a relatively novel approach, adding to the knowledge in this field, and offering new insights in the approach of constructing an FI. Moreover, the emphasis on the meaning of the items provided for by the GRM possibly leads to a more adequate measurement of frailty.

Nevertheless, some study limitations need consideration. First, the new deficits were included based on face validity, and existing ones were selected from previously validated scales [24, 37, 38]. Even though the FI-HM37 is similar to previously constructed and validated FIs [16–18], and frailty scores systematically increase with age, follow-up research to further establish its predictive and concurrent validity will be useful. Second, the deficits in the DPHM are selected by the Community Health Services, Statistics Netherlands and the National Institute for Public Health and the Environment, which limited the choice of deficits to be included in the current study. For example, the social domain was measured only by the De Jong Gierveld scale for social and emotional loneliness, and the psychological domain only by the K10 scale for anxiety/depression and the Pearlin Mastery scale [24, 37, 38]. Social and psychological frailty, however, may encompass more than loneliness, anxiety or depression and mastery. Moreover, other health domains, such as the cognitive domain, were not accounted for in the data of the DPHM and could therefore not be included in the FI-HM37. Cognitive frailty is increasingly gaining attention in frailty research [3], and results of a recent systematic review and meta-analysis showed that older adults with cognitive frailty are at higher risk of several adverse health outcomes than older adults without cognitive frailty [41]. In light of these findings, the cognitive frailty domain would have been an interesting addition to the current study.

The findings in this study identify important implications for the field of epidemiological research, on public health and for FI development methods. Notably, we have shown that even when a health survey does not include an established frailty instrument, frailty can be operationalized using a deficit accumulation approach. Using the DPHM to provide information about frailty levels of persons or groups in society could inform policymakers on different governance levels regarding frailty management as well as frailty prevention or postponement, both for overall frailty as well as for specific frailty domains. Furthermore, it would inform epidemiologists about frailty levels in The Netherlands, and about groups in the population that are frail or face the risk of becoming frail. Such type of research would increase the usability of the DPHM e.g. for determining risk groups among the older population. Besides, the rigorous examination of the separate health deficits possibly led to an FI that approaches the concept of frailty more accurately than an FI lacking psychometric analysis. The FI-HM37 resulted from exclusions of five deficits that did not pass well established psychometric measurement criteria, making the FI-HM37 a more concise scale [22]. The remaining deficits cover several health domains, which is inherent to an FI [15], and the health deficits show variation in severity of deficit (probability of respondents reporting a health deficit), indicating that the health deficits cover the continuum of frailty [34]. Furthermore, the FI-HM37 shows high scale reliability. First results on external validation by assessing predictive validity of the FI-HM37 seem positive, which we will report in a separate paper. These results indicate that the FI-HM37 is a concise scale with favorable measurement properties that could possibly facilitate the determination of frailty levels in the Dutch population.

## Conclusions

To conclude, an FI with 37 psychometrically strong items was developed using the DPHM, hereby operationalizing frailty aimed at population measurement. The study has shown both the possibility of using an existing national health survey for measuring frailty, as well as the importance of deficit selection during FI construction. Taking the DPHM as the basis for the construction of the FI-HM37, is promising for further health and epidemiological frailty research, offering a solid base for measuring frailty levels in Dutch older adults living in the community. By estimating frailty levels on a nationwide scale, the current study adds to the need of gaining insight in the frailty status on population level. These results might serve as a first step in contributing to governmental measures for prevention or postponement of frailty. More research is recommended to investigate predictive and concurrent validity of the FI-HM37, e.g., to investigate regional or group differences.

## Data Availability

Results are based on analyses of non-public microdata from Statistics Netherlands by the authors. Under certain conditions, these microdata are accessible for statistical and scientific research. For further information: microdata@cbs.nl.
